# Study protocol for the development and validation of a clinical prediction tool to estimate the risk of 1-year mortality among hospitalized patients with dementia

**DOI:** 10.1186/s41512-024-00168-2

**Published:** 2024-03-19

**Authors:** Michael Bonares, Stacey Fisher, Kieran Quinn, Kirsten Wentlandt, Peter Tanuseputro

**Affiliations:** 1 Department of Medicine, Sunnybrook Health Sciences Centre, 2075 Bayview Avenue, Toronto, ON M4N 3M5 Canada; 2 Department of Medicine, University of Toronto, Toronto, ON Canada; 3 Ottawa Hospital Research Institute, Ottawa, ON Canada; 4 Department of Medicine, Sinai Health System, Toronto, ON Canada; 5 ICES Toronto, Toronto, ON Canada; 6 Department of Supportive Care, University Health Network, Toronto, ON Canada; 7 Department of Family and Community Medicine, University of Toronto, Toronto, ON Canada; 8 Department of Medicine, University of Ottawa, Ottawa, ON Canada; 9 Bruyère Research Institute, Ottawa, ON Canada; 10 ICES Ottawa, Ottawa, ON Canada

**Keywords:** Dementia, Hospitals, Prognosis, Mortality, Advance care planning, Palliative care

## Abstract

**Background:**

Patients with dementia and their caregivers could benefit from advance care planning though may not be having these discussions in a timely manner or at all. A prognostic tool could serve as a prompt to healthcare providers to initiate advance care planning among patients and their caregivers, which could increase the receipt of care that is concordant with their goals. Existing prognostic tools have limitations. We seek to develop and validate a clinical prediction tool to estimate the risk of 1-year mortality among hospitalized patients with dementia.

**Methods:**

The derivation cohort will include approximately 235,000 patients with dementia, who were admitted to hospital in Ontario from April 1st, 2009, to December 31st, 2017. Predictor variables will be fully prespecified based on a literature review of etiological studies and existing prognostic tools, and on subject-matter expertise; they will be categorized as follows: sociodemographic factors, comorbidities, previous interventions, functional status, nutritional status, admission information, previous health care utilization. Data-driven selection of predictors will be avoided. Continuous predictors will be modelled as restricted cubic splines. The outcome variable will be mortality within 1 year of admission, which will be modelled as a binary variable, such that a logistic regression model will be estimated. Predictor and outcome variables will be derived from linked population-level healthcare administrative databases. The validation cohort will comprise about 63,000 dementia patients, who were admitted to hospital in Ontario from January 1st, 2018, to March 31st, 2019. Model performance, measured by predictive accuracy, discrimination, and calibration, will be assessed using internal (temporal) validation. Calibration will be evaluated in the total validation cohort and in subgroups of importance to clinicians and policymakers. The final model will be based on the full cohort.

**Discussion:**

We seek to develop and validate a clinical prediction tool to estimate the risk of 1-year mortality among hospitalized patients with dementia. The model would be integrated into the electronic medical records of hospitals to automatically output 1-year mortality risk upon hospitalization. The tool could serve as a trigger for advance care planning and inform access to specialist palliative care services with prognosis-based eligibility criteria. Before implementation, the tool will require external validation and study of its potential impact on clinical decision-making and patient outcomes.

**Trial registration:**

NCT05371782.

## Background

### Advance care planning in dementia

Patients with dementia and their caregivers are faced with difficult healthcare decisions, especially at advanced stages of disease. These decisions are made more difficult by the inevitability that patients with dementia will lose capacity to make them [1]. Early engagement in advance care planning facilitates timely shared decision-making between a patient, their substitute decision maker, and their healthcare provider. There is evidence for the benefit of advance care planning among dementia patients. A systematic review of advance care planning in this patient population demonstrated an association with an increase in documentation of advance directives, the number of discussions about goals of care, and the receipt of goal-concordant care [2]. Despite these benefits, there is a deficiency of advance care planning in this patient population [3, 4]. This may be caused by a variety of barriers, including uncertainty about the optimal timing of advance care planning, difficulty in planning for an uncertain future, patients’ and caregivers’ lack of knowledge about dementia, difficulty in the assessment of decisional capacity among dementia patients, and the possibility that preferences may change over time [5]. A lack of advance care planning may enable decisions that result in frequent acute care utilization [6–12], the pursuit of life-prolonging interventions [13–15], and absent or delayed access to specialist palliative care [11, 16–19] in the advanced stages of disease. By identifying individuals with dementia who have a high risk of mortality, a prognostic tool could serve as a prompt to healthcare providers to initiate advance care planning among patients and their caregivers. It could also trigger a referral to specialist palliative care services if needs are complex. This could result in the receipt of care that is concordant with a patient’s goals.

### Prognostic tools among hospitalized patients with dementia

The hospital is a pragmatic setting in which a prognostic tool among patients with dementia could be implemented. Patients with dementia are frequently hospitalized, especially as they approach the end of life [6–12], such that a hospital-based prognostic tool could be applied to a high proportion of this patient population. In addition, hospitalization may represent a significant point of inflection among patients with dementia. Specifically, hospitalized patients with dementia have a higher risk of mortality and readmission than patients without dementia [20]. In addition, hospitalizations among patients with dementia who were living at home may result in a discharge to a long-term care facility [21, 22]. Therefore, hospitalization could serve as a touchpoint to have a discussion about the expected disease trajectory, informed by the output of a prognostic tool.

There have been 3 attempts to develop a prognostic tool among hospitalized patients with dementia [23–25]. They have limitations that decrease their clinical utility. First, the studies have methodological weaknesses regarding variable selection [24], specification of continuous variables [24, 25], and handling of missing values [25]. Second, the tools had a limited number of predictor variables, and none of them included non-linear or interaction terms. Therefore, they may not be sufficiently complex to capture the nuances of mortality risk prediction in this patient population. Third, their performance was assessed in terms of discrimination, which ranged from poor to acceptable. However, it was not or inadequately assessed and/or reported in terms of calibration, which provides an indication of a prognostic tool’s ability to make accurate predictions for new patients. All of the models were judged to be at high risk of bias based on the Prediction model Risk Of Bias ASsessment Tool (PROBAST) [26]. Otherwise, one study included clinically meaningful variables, but ones that were collected through a comprehensive geriatrics assessment, which limits its utility [23]. Another study sought to predict mortality among only patients with dementia who underwent surgery for a hip fracture, which limits its generalizability [25]. None of these models have been externally validated or are used in practice to our knowledge.

### Objectives

This study seeks to develop and validate a clinical prediction tool to estimate the risk of 1-year mortality among a cohort of patients with dementia hospitalized in Ontario from April 1st, 2009, to March 31st, 2019. The model will include a comprehensive list of clinically meaningful variables that are routinely collected in population-level healthcare administrative databases, and non-linear and interaction terms. In addition, it will comprise an inclusive cohort of hospitalized patients with dementia, not limiting it to those with specific complications. Model performance will not only be assessed in terms of predictive accuracy and discrimination*,* but also in terms of calibration in the validation cohort and in predefined subgroups of importance to clinicians and policymakers. Finally, the protocol will adhere to recommendations for the development [27] and reporting [28, 29] of prognostic studies.

## Methods/design

### Study design

The model will be derived and validated using population-level data in the healthcare administrative databases at ICES in Ontario, Canada. Ontario is Canada’s most populous province, comprising 14.8 million residents [30]. This model, which seeks to predict mortality among hospitalized patients with dementia, would be well served by population-based data in Ontario, since the high rates of both acute care utilization [31] and mortality [32] among patients with dementia have been established therein. Patients or the public were not involved in the design and will not be involved in the conduct, reporting, or dissemination of this study. However, they will be involved in the implementation of the clinical prediction tool.

In Ontario, healthcare is administered through a primarily public system, which is accessible to all individuals by means of a universal health insurance program. ICES houses multiple databases that include data on all individuals in Ontario who are eligible for the program. Of most interest to this study is the Discharge Abstract Database (DAD), which is maintained by the Canadian Institute for Health Information [33], and from which predictor variables will be identified. This database includes information on individuals who have been admitted to any acute care facility in Ontario, which has been collected from discharge summaries by trained abstractors. The presence of the DAD in all other provinces and territories of Canada lends itself to the application of this tool beyond Ontario.

### Eligibility criteria

Individuals with dementia will be identified using a validated algorithm to identify cases based on a combination of hospital codes, physician claims, and prescribed medications that are specific to dementia [34]. This algorithm was validated and found to be sufficiently sensitive (79.3%) and highly specific (99.1%) [34]. Individuals will be included if they were hospitalized at least once from April 1st, 2009, to March 31st, 2019. Exclusion criteria will be age < 65 years at the time of admission to exclude phenotypes of early-onset dementia, which may be informed by a different profile of predictor variables; no age or sex at the time of admission; ineligibility for universal health insurance at the time of admission or for ≥ 3 months before admission; and death recorded as having happened before or at the time of admission. If a patient was admitted more than once during the 10-year accrual window, then only the first admission will be included to avoid survival bias.

### Outcome

The outcome variable is mortality within 1 year of admission, which will be modelled as a binary variable. This will be ascertained through linkage to a population-based registry, the Registered Persons Database, which is maintained by the Ontario Ministry of Health and Long-Term Care. This contains a historical listing of all healthcare numbers that have been issued under Ontario’s universal health insurance program, demographic information (i.e., age, sex, postal code), and vital status, including date of death.

### Sample size

The cohort includes approximately 298,000 patients. It will be split temporally into derivation and validation cohorts. The derivation cohort will comprise about 235,000 patients (~ 80%), hospitalized from 2009 to 2017, and the validation cohort will comprise about 63,000 patients (~ 20%), hospitalized from 2018 to 2019.

A major consideration in prediction modelling is overfitting, which happens in the context of including a disproportionately high number of degrees of freedom in the model relative to the number of events in the cohort. For a binary logistic regression model, the number of participants in the smaller of the two outcome groups should be at least 10 times higher than the number of degrees of freedom in the model [35]. The derivation cohort includes about 82,000 deaths within 1 year of admission. Therefore, the maximum number of degrees of freedom is expected to be significantly higher than the number in the prespecified model, such that overfitting is unlikely. We calculated the minimum sample size using the *pmsampsize* package in R according to the approach proposed by Riley et al. [36, 37]. Assuming a conservative c-statistic of 0.77 [23], an event proportion of 0.35, 205 parameters, and a shrinkage factor of 0.90, the minimum recommended sample size is 8251. The expected sample size surpasses this recommendation, and as a result, we anticipate having a sufficient sample size. It has also been recommended that there be at least 100 events and 100 non-events in a validation cohort [38]. The validation cohort includes about 21,000 deaths within 1 year of admission. Therefore, we will have the minimum number of events and non-events.

### Analysis plan

The analysis plan was informed by guidelines for regression modelling [39] and clinical prediction models [35] after accessing the derivation dataset but before model fitting or analyses of predictor-outcome associations. Key considerations of the analysis plan are full prespecification of predictor variables and avoidance of data-driven variable selection, and use of flexible functions to model continuous predictors. Analyses will be conducted using SAS Enterprise Guide V.9.4. The reporting of the model will be guided by the TRIPOD statement for multivariable prediction models for prognosis [29].

#### Identification of predictors

The predictor variables have come from 3 sources. First, variables used in the modified Hospital One-Year Mortality (mHOMR), most of which are available in the DAD, will be included in the initial model. This prognostic tool was established to predict mortality 1 year after hospitalization [40]. Though it was not specifically created for patients with dementia, we judged it to serve as an ideal framework on which to build our model for several reasons. First, it was derived from and internally validated in Ontario, demonstrating high discrimination and calibration [40], and has been externally validated in a temporally distinct population in Ontario and two geographically distinct populations in Alberta, Canada, and Boston, Massachusetts, demonstrating high discrimination and calibration [41]. Second, it was judged to be both a feasible [42] and acceptable [43] tool for mortality risk prediction by patients, caregivers, and healthcare providers. Third, a high mHOMR score has been associated with unmet palliative care needs [44]. Finally, a version of mHOMR has been successfully implemented in several Ontario hospitals.

Second, we identified variables in existing prognostic indices [23–25] or that have an established association with mortality based on etiological studies [45–57] among hospitalized patients with dementia. The existing prognostic indices were identified through a systematic review using a single electronic database (PubMed), in addition to hand-searching of each of their reference lists. The etiological studies were identified in a recent scoping review of prognostic factors in dementia [58]. We then identified, where possible, correlates of these variables in the DAD. Finally, we reviewed all variables in the DAD to identify those that, based on subject-matter expertise, could inform mortality risk prediction in this patient population but are not present in either the mHOMR or the literature. Specifically, our research team includes clinical expertise in internal medicine, family medicine, public health, and palliative medicine. Since the ultimate objective is to have the clinical tool generate a risk of 1-year mortality at the time of admission, predictor variables that would not be known at the time of admission (e.g., admitting diagnosis, length of stay) were not considered. Variables with narrow distributions or insufficient variation will be excluded (e.g., binary variables for which one level has few or no observations). Variables with a high proportion of missing values (> 50%) will be excluded. While multiple imputation (see Missing data) may perform well if a variable has > 90% missingness [59], a variable with > 50% missing may be of poor quality and result in bias. A total of 87 predictor variables were identified and categorized as follows: sociodemographic factors (3), comorbidities (51), previous interventions (8), functional status (11), nutritional status (7), admission information (4), previous health care utilization (3) (Table 1). For comorbidities and previous interventions, a 3-year lookback window will be used, which is in keeping with the most recent iteration of the mHOMR [60].

**Table 1:** Prespecification of predictor variables for a mortality risk prediction tool among hospitalized patients with dementia with initial degrees of freedom allocation

Variable	Scale	Initial variable specification	Degrees of freedom
Sociodemographic factors			
Age	Continuous	5-knot spline: valid range: 65 to 105 years	4
Sex	Categorical	Male; female	1
Living status	Categorical	5 categories: home, independent; home, home care; nursing home; rehabilitation; chronic hospital	4
Comorbidities			
Cerebrovascular disease	Categorical	Yes; no	1
Heart failure	Categorical	Yes; no	1
Cardiovascular disease	Categorical	Yes; no	1
Arrhythmia	Categorical	Yes; no	1
Cardiac arrest	Categorical	Yes; no	1
Chronic obstructive pulmonary disease	Categorical	Yes; no	1
Asthma	Categorical	Yes; no	1
Pneumonia	Categorical	Yes; no	1
Pneumonitis	Categorical	Yes; no	1
Adult respiratory distress syndrome	Categorical	Yes; no	1
Interstitial lung disease	Categorical	Yes; no	1
Respiratory failure	Categorical	Yes; no	1
Gastrostomy site infection	Categorical	Yes; no	1
Liver cirrhosis	Categorical	Yes; no	1
Urinary tract infection	Categorical	Yes; no	1
Fecal incontinence	Categorical	Yes; no	1
Urinary incontinence	Categorical	Yes; no	1
Renal failure	Categorical	Yes; no	1
Decubitus ulcer	Categorical	Yes; no	1
Parkinson’s disease	Categorical	Yes; no	1
Multiple sclerosis	Categorical	Yes; no	1
Osteoporosis	Categorical	Yes; no	1
Fracture	Categorical	Yes; no	1
Malignancy			
Lip, oral cavity, pharynx	Categorical	Yes; no	1
Digestive organs	Categorical	Yes; no	1
Respiratory, intrathoracic organs	Categorical	Yes; no	1
Skin	Categorical	Yes; no	1
Mesothelium, soft tissue	Categorical	Yes; no	1
Breast	Categorical	Yes; no	1
Female genital organs	Categorical	Yes; no	1
Male genital organs	Categorical	Yes; no	1
Urinary tract	Categorical	Yes; no	1
Central nervous system	Categorical	Yes; no	1
Endocrine glands	Categorical	Yes; no	1
Bone	Categorical	Yes; no	1
Unspecified	Categorical	Yes; no	1
Blood	Categorical	Yes; no	1
Multiple	Categorical	Yes; no	1
Sepsis	Categorical	Yes; no	1
Diabetes mellitus	Categorical	Yes; no	1
Hypertension	Categorical	Yes; no	1
Peripheral arterial disease	Categorical	Yes; no	1
Shock	Categorical	Yes; no	1
Mood disorder	Categorical	Yes; no	1
Schizophrenia	Categorical	Yes; no	1
Complication from benzodiazepine	Categorical	Yes; no	1
Complication from antipsychotic	Categorical	Yes; no	1
Fall	Categorical	Yes; no	1
vChronic kidney disease	Categorical	Yes; no	1
Rheumatoid arthritis	Categorical	Yes; no	1
Polypharmacy	Categorical	Yes; no	1
Previous interventions			
Heart resuscitation	Categorical	Yes; no	1
Mechanical ventilation lasting ≥ 96 h	Categorical	Yes; no	1
Mechanical ventilation lasting < 96 h	Categorical	Yes; no	1
Dialysis	Categorical	Yes; no	1
Cardioversion	Categorical	Yes; no	1
Chemotherapy	Categorical	Yes; no	1
Radiotherapy	Categorical	Yes; no	1
Tracheostomy	Categorical	Yes; no	1
Functional status			
Hospital Frailty Risk Score	Continuous	5-knot spline: valid range 0 to 99	4
Dysphasia and aphasia	Categorical	Yes; no	1
Dysarthria and anarthria	Categorical	Yes; no	1
Dysphagia	Categorical	Yes; no	1
Dependence on wheelchair	Categorical	Yes; no	1
Blindness	Categorical	Yes; no	1
Hearing loss	Categorical	Yes; no	1
Hospital-based occupational therapy consultation in past 3 years	Categorical	Yes; no	1
Hospital-based physiotherapy consultation in past 3 years	Categorical	Yes; no	1
Hospital-based social worker consultation in past 3 years	Categorical	Yes; no	1
Hospital-based speech language pathology consultation in past 3 years	Categorical	Yes; no	1
Nutritional status			
Weight	Continuous	5-knot spline	4
Height	Continuous	5-knot spline	4
Parenteral nutrition	Categorical	Yes; no	1
Feeding tube	Categorical	Yes; no	1
Anorexia	Categorical	Yes; no	1
Abnormal weight loss	Categorical	Yes; no	1
Volume depletion	Categorical	Yes; no	1
Admission information			
Admitting service	Categorical	15 categories: General internal medicine; Cardiology; Gastroenterology; Palliative care; Medical oncology; General surgery; Cardiovascular surgery; Neurosurgery; Orthopedic surgery; Plastic surgery; Thoracic surgery; Trauma; Urology; Gynecology; Psychiatry	14
Admission urgency	Categorical	3 categories: elective; urgent, without ambulance; urgent, with ambulance	2
Admission directly to ICU	Categorical	Yes; no	1
Urgent 30-day readmission	Categorical	Yes; no	1
Previous healthcare utilization			
ED visits in past 12 months	Ordinal	7 categories: 0, 1, 2, 3, 4, 5, 6 +	6
Admissions by ambulance in past 12 months	Ordinal	4 categories: 0, 1, 2, 3 +	3
Hospital-based palliative care in past 3 years	Categorical	Yes; no	1

Age will be interacted with all variables except those listed under sociodemographic factors, admission information, and previous health care utilization; renal failure will be interacted with dialysis; each site of malignancy will be interacted with chemotherapy

*ICU* Intensive care unit, *ED* Emergency department

The model will include age interactions with variables that represent comorbidities and previous interventions, since the association of these variables with mortality may vary with age. It will also include interactions of diseases with corresponding treatments, including kidney dysfunction with dialysis, and malignancy with chemotherapy.

#### Data cleaning and coding of predictors

All variables will be evaluated for potentially invalid values. Specifically, continuous variables will be inspected using descriptive statistics and boxplots. Categorization of continuous variables will be avoided to prevent loss of predictive information. Categorical variables will be inspected using frequency distributions. Should invalid values be identified, then they will be corrected, if possible, and otherwise, set to missing. All data cleaning and coding will be performed before analyzing predictor-outcome associations.

#### Missing data

Missing values will be imputed with multiple imputation methods. This is in contrast to complete case analysis, which suffers from inefficiency and selection bias [35]. Multiple imputation is performed in 3 main steps [61, 62]. First, an imputation model is established to impute missing values using random draws from the conditional distribution of the missing variable based on all the other variables in the analytic model. This imputation is performed *M* number of times, which is contingent on the proportion of missing values. It has been recommended that *M* be ≥ 100 × *p*, where *p* is the proportion of missing values [63]. This step creates *M* number of imputation datasets, each with different imputed values, which accounts for the uncertainty associated with imputation. Second, logistic regression will be performed in each of the *M* imputation datasets, each resulting in different parameter estimates. Finally, the parameter estimates will be combined to generate the final parameter estimates of the analytic model [64]. This will be performed using the MI [65] and MIANALYZE [66] functions in SAS.

#### Model specification

Continuous variables will be flexibly modelled using restricted cubic splines, with knots placed at fixed quantiles of distribution (e.g., in a five-knot spline function, 5th, 27.5th, 50th, 72.5th, and 95th). Categorical variables with levels that include a low number of observations will be combined with others. Interactions will be restricted to linear terms. The initial model includes a total of 205 of degrees of freedom, 111 of which are of main effects and 94 of which are of interaction effects.

The predictive potential of each variable will be determined by a method described by Harrell [39]. Specifically, a general effects model will be created that includes all predictor variables. The degrees of freedom of each variable will be subtracted from its *χ*(2) statistic to level the playing field given that the variables have varying degrees of freedom. These values will be plotted from highest to lowest. Variables with higher predictive potential will retain their initial degrees of freedom; whereas those with lower predictive potential will be modelled as simple linear terms or have their infrequent categories combined. This does not increase the type 1 error rate since all predictor variables will be retained in the final model regardless of their strength of association with the outcome variable.

#### Model estimation

The model will be estimated using a binary logistic regression. All predictor variables will be centered about their means. Multi-collinearity will be assessed using variable clustering using the VARCLUS function in SAS [67]. In addition, the variance inflation factor (VIF) will be calculated for each predictor variable. Predictors with a VIF > 2.5 will be judged to be potentially collinear. These methods will be used to understand the model with more clarity though will not result in further model specification.

Overfitting will be assessed visually using the calibration plot (see Assessment of performance), and statistically using the heuristic shrinkage estimator [68]. If the estimator is less than 0.9, then this suggests the presence of overfitting that requires adjustment, including shrinkage of the parameter estimates. This is unlikely considering prespecification of the model, and the low number of degrees of freedom in the model relative to the high number of events in the cohort. We intend to have the model provide an automatic calculation of mortality risk, not to have it used manually to calculate risk (see Model presentation). Therefore, predictive accuracy will be prioritized over parsimony, and a reduced model will not be estimated.

The model will be developed and validated using temporally split samples; however, the final regression coefficients will be based on the full sample. The final model will have the same specifications as the derivation model.

#### Assessment of performance

The model’s performance will be assessed using temporal validation, a type of internal validation. Whereas internal validation using random splitting or resampling (i.e., bootstrapping, cross-validation) evaluates reproducibility, it does not evaluate transportability. In contrast, temporal validation evaluates transportability since the cohort is temporally distinct from the derivation cohort [69–71].

The final model’s performance will be assessed and reported using measures of predictive accuracy, discrimination, and calibration in the validation cohort. Predictive accuracy will be measured by means of Nagelkerke’s *R*^*2*^[72]. Discrimination represents the model’s ability to distinguish between those who have and those who do not have the outcome. It will be measured using the c-statistic, which is equivalent to the area under the receiver operating characteristic curve when the outcome is binary. Calibration represents the model’s ability to generate predictions of the probability of death that mirror the observed probability of death. It will be assessed visually by means of a calibration plot, generated by regressing the outcome variable on the predicted risk of mortality. The slope and intercept of the calibration curve will be calculated; a model with a slope of 1 and intercept of 0 is perfectly calibrated. The clinically relevant standard of calibration is defined as less than a 20% difference between observed and predicted probabilities with an event rate of at least 5% [73]. Calibration will also be evaluated within predefined subgroups of importance to clinicians and policymakers (e.g., groups defined by age, sex, living status, and comorbid disease). Calibration will not be assessed via statistical means, namely the Hosmer–Lemeshow statistic, since it has been shown to perform poorly in large samples [38] and to have insufficient power to detect overfitting [39].

## Discussion

### Model presentation

The final regression model, based on the total cohort, will be presented using beta coefficients and corresponding standard errors.

**Fig. 1:**
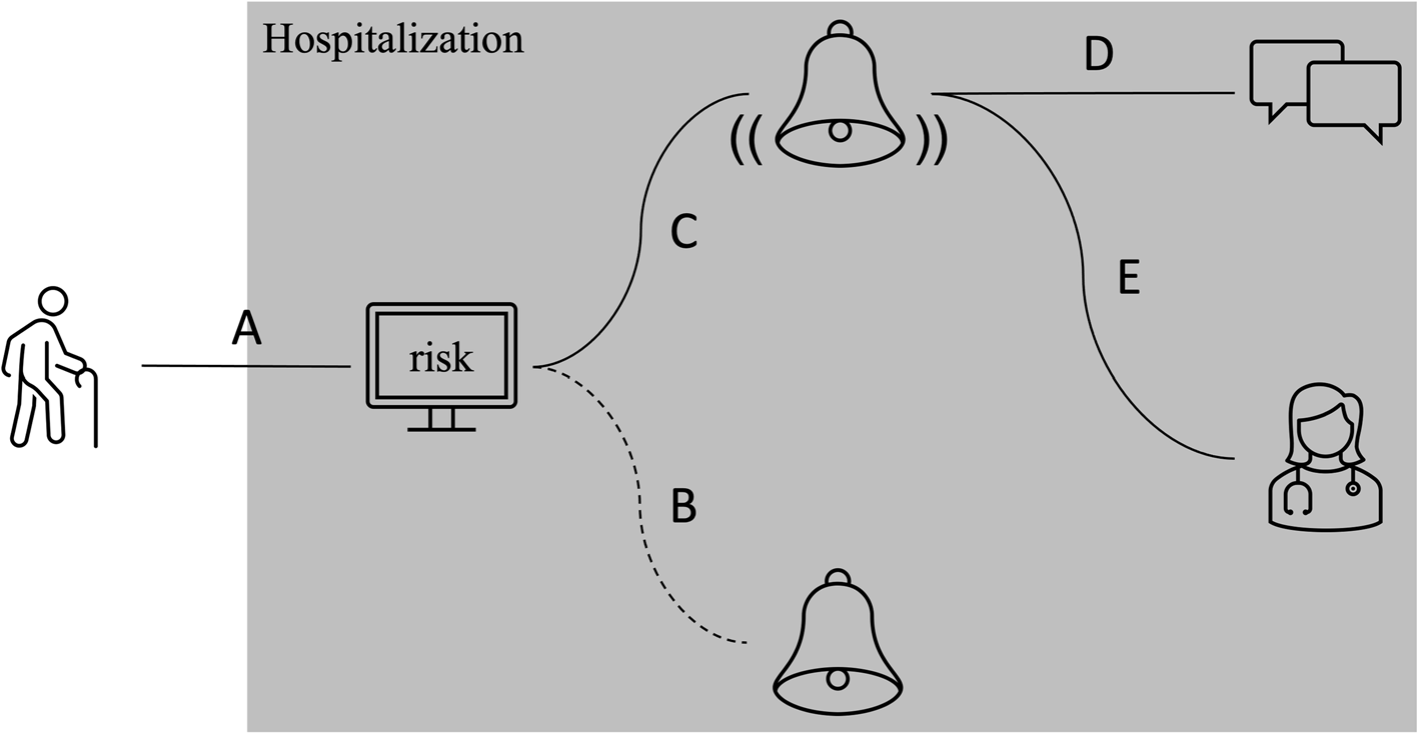
**A** A patient with dementia is hospitalized, at which point a risk of 1-year mortality is automatically calculated by the computer. **B** If the risk of mortality is below a user-defined threshold, then the admitting team is not alerted. The threshold is based on the capacity of medical and specialist palliative care teams to act on the alert. **C** If the risk of mortality is above the threshold, then the admitting team is alerted. **D** The alert could prompt the admitting team to initiate advance care planning (primary palliative care). **E** The alert could prompt the admitting team to refer to specialist palliative care if needs are complex

The regression formula of the model will be disseminated on a publicly accessible website, www.projectbiglife.com, which houses existing clinical prediction tools developed by our team. Because of the complexity of the model, the tool will not be used manually. Rather, it will be integrated into the electronic medical records of hospitals, such that the risk of 1-year mortality will be automatically calculated by the computer upon admission. For example, upon admission, based on routinely collected information in a hospital’s data warehouse, the risk of 1-year mortality will be automatically calculated for a patient with dementia. If the risk exceeds a threshold, then the admitting team will be alerted. The threshold will be based on the capacity of medical, surgical, and specialist palliative care teams to provide care (e.g., a high threshold if the teams have a limited capacity). Equipped with and impelled by this information, they will be able to engage in discussions about goals of care (primary palliative care) or make a referral to the specialist team if needs are complex (Fig. 1). This implementation is similar to that of the mHOMR in Ontario.

### Contribution of this study

This tool has the potential to inform health policy, research, and clinical practice. First, it could inform eligibility criteria for specialist palliative care services, including those for hospice. These criteria, which are usually based on functional status and estimated prognosis, may presently be restrictive to patients with dementia and other non-malignant diseases [74], which are characterized by an undefined “terminal” phase of disease [75]. For example, a tool that was intended to predict 6-month mortality among nursing home residents with advanced dementia [76] performed better than Medicare hospice eligibility guidelines [77]. This demonstrates the potential limitations of the rules that presently govern how palliative care is accessed, and the promise of a prognostic tool to mitigate these limitations by more sufficiently informing the decision to access palliative care. Second, the tool could identify cohorts of patients with dementia that could most benefit from palliative care interventions. This could facilitate a more targeted selection of participants in both research trials and quality improvement initiatives.

Finally, the tool could minimize the barrier of prognostic uncertainty [75, 78–80] of hospitalized patients with dementia to palliative care. That is, if a patient is found to have a high risk of 1-year mortality, then this could prompt their provider to pursue palliative care. This would appear to go against the widely accepted knowledge that the pursuit of palliative care be based on needs rather than on prognosis [81, 82]. In practice, a prognostic tool would not supplant the needs-based approach but would complement it. For example, a clinician who identifies palliative care needs in a patient with dementia, paralyzed by prognostic uncertainty and falsely reassured by the possibility of “more time” [80], may not refer to specialist palliative care. A prognostic tool that identifies the patient as high risk for mortality could empower the clinician to do so. In concert, the assessment of needs and the estimation of prognosis would identify patients who require specialist palliative care intervention most urgently; this approach would accommodate the current limitations in the capacity of specialist palliative care providers to deliver services en masse.

### Limitations

We sought to include primarily predictor variables available in the DAD. Though this database includes the vast majority of predictors considered in previous etiological studies and existing prognostic indices, it does not include variables available in other datasets that could inform mortality risk prediction among patients with dementia, namely, sociodemographic information (e.g., immigration status, race, education level, income quantile, rurality), functional status (e.g., activities of daily living, instrumental activities of daily living), and nutritional status (e.g., body mass index). The absence of these variables may limit the predictive accuracy of the model. However, including primarily variables in the DAD facilitates external validation of the model in other Canadian jurisdictions, wherein linkage between the DAD and other datasets is not readily possible. The tool may not be able to be implemented in healthcare systems outside of Canada if it is not currently possible to draw on data from a hospital’s data warehouse to enter into a prediction model. This limits its applicability outside of Canada.

Based on previous knowledge of the DAD, we expect that some prespecified predictor variables will have a high proportion of missing values (e.g., height and weight). We intend to pursue multiple imputation to infer missing values. However, should the values be missing not at random, then the variables may be refractory to multiple imputation and will be excluded from the model despite the possibility that they could inform mortality risk prediction in this patient population. Indeed, substantial missingness would suggest that the variables may not commonly be captured in the discharge summaries of hospitalized patients with dementia. Therefore, the inclusion of these variables in the model would limit its application. Finally, even if the analysis demonstrates acceptable performance of the model, it will require external validation to confirm its generalizability. Thereafter, implementation studies will be required to determine the potential impact of the tool on clinical decision-making, patient outcomes, and/or healthcare costs.

### Conclusion

A clinical prediction tool that provides personalized estimates of mortality among hospitalized patients with dementia has the potential to prompt advance care planning, increase access to primary and specialist palliative care, and ultimately, to facilitate receipt goal-concordant healthcare. Similar to the mHOMR, which has been implemented in several Ontario hospitals, this model could be integrated into electronic medical records as an automated tool for mortality risk prediction as soon as a patient with dementia is hospitalized, which could inform their care both during admission and upon discharge.

This model advances the work done by previous investigators by creating a population-level prognostic tool among hospitalized patients with dementia that is sufficiently complex to reflect the intricacies of mortality risk prediction in this patient population. The analysis plan will be informed by guidelines for regression modelling and clinical prediction models, and reporting of the model will adhere to the TRIPOD statement for multivariable prediction models for prognosis. Although the model will be internally (temporally) validated, it will require external validation and investigation of its potential impact on clinical decision-making, patient outcomes, and healthcare costs before implementation.

## Data Availability

The dataset used for this study will be held securely in a de-identified form at ICES. While data sharing agreements prohibit ICES from making the dataset publicly available, access may be granted to individuals who meet prespecified criteria for confidential access, available at www.ices.on.ca/DAS.

## Abbreviations

DAD: Discharge Abstract Database
mHOMR: Modified Hospital One-Year Mortality Risk
VIF: Variance inflation factor
